# Statin Treatment on Cardiovascular Risk After Retinal Artery Occlusion: A Historical Cohort Study

**DOI:** 10.1007/s44197-023-00143-y

**Published:** 2023-08-12

**Authors:** Joonsang Yoo, Jimin Jeon, Joo Youn Shin, Minyoul Baik, Jinkwon Kim

**Affiliations:** 1 Department of Neurology, Yongin Severance Hospital, Yonsei University College of Medicine, 363 Dongbaekjukjeon-Daero, Giheung-Gu, Yongin-Si, Gyeonggi-Do 16995 Republic of Korea; 2 Department of Ophthalmology, The Institute of Vision Research, Yongin Severance Hospital, Yonsei University College of Medicine, Yongin, Korea

**Keywords:** Cardiovascular disease, Myocardial infarction, Population-based study, Retinal artery occlusion, Statin, Stroke

## Abstract

**Introduction:**

Retinal artery occlusion (RAO) is a major cause of acute visual loss and patients with RAO have an increased risk for subsequent cardiovascular events. However, there is little evidence of whether the use of statins is associated with the prevention of cardiovascular events in patients with RAO. We investigated whether statin treatment in patients with RAO is associated with a lower risk of cardiovascular events.

**Methods:**

This study was a historical cohort study with nested case–control analysis. Using the nationwide health insurance claims database in Korea, we retrospectively established a cohort of newly diagnosed RAO patients without prior cardiovascular events between January 2008 and March 2020. We defined the case group as those who had cardiovascular events (stroke or myocardial infarction) and the control group as RAO patients without primary outcome matched by sex, age, comorbidities, and duration of follow-up (1:2 incidence density sampling). Conditional logistic regression was performed.

**Results:**

Among 13,843 patients newly diagnosed with RAO, 1030 patients had cardiovascular events (mean follow-up period of 6.4 ± 3.7 years). A total of 957 cases were matched to 1914 controls. Throughout the study period, the proportion of patients taking statin was less than half. Statin treatment after RAO was associated with a low risk of cardiovascular events (adjusted OR, 0.637; 95% CI 0.520–0.780; *P* < 0.001). A longer duration of statin exposure was associated with a lower cardiovascular risk.

**Conclusions:**

In patients with newly diagnosed RAO, treatment with statins, particularly long-term use, was associated with a low risk of future cardiovascular events.

**Supplementary Information:**

The online version contains supplementary material available at 10.1007/s44197-023-00143-y.

## Introduction

Retinal artery occlusion (RAO) is an ophthalmic emergency characterized by the sudden onset of monocular visual loss, which can result in permanent visual disability. With an incidence of 1–2 cases per 100,000 person-years, RAO is primarily caused by impaired arterial blood flow to the retinal circulation leading to ischemic damage [1, 2]. The mechanism of atherosclerosis or thromboembolism-related blockage of the retinal artery is essentially similar to that of ischemic stroke, in which brain tissue damage occurs due to impairment of cerebral blood flow [3, 4]. RAO and ischemic stroke share various vascular risk factors, such as old age, hypertension, diabetes mellitus, dyslipidemia, smoking, atherosclerosis, and atrial fibrillation [5, 6]. Furthermore, acute ischemic stroke is often concomitantly identified as RAO is diagnosed [7, 8].

Epidemiologic evidence has established that patients with RAO are at an increased risk of subsequent cardiovascular events that is comparable to the risk associated with ischemic stroke [8–10]. Given the high mortality and socioeconomic burden of cardiovascular complications, it is crucial to establish optimal preventive strategies for patients with RAO who are at high risk. Statins, also called HMG-CoA reductase inhibitors, are a class of lipid-lowering agents and are among the most commonly prescribed drugs worldwide. Evidence from multiple clinical trials has demonstrated that statin therapy is effective at reducing cardiovascular events in patients with established atherosclerotic cardiovascular disease and individuals at high baseline risk of cardiovascular disease [11]. Considering the atherothrombotic pathogenesis and high cardiovascular risk in RAO patients, statin treatment after RAO may effectively reduce future cardiovascular events [12]; hence, they are often recommended in clinical practice [13]. However, there is little evidence of the preventive role of statins in patients with RAO. Using a nationwide health claim database, we investigated whether statin treatment in RAO patients was associated with a lower risk of subsequent cardiovascular events.

## Materials and Methods

### Study Design and Data Source

This study was a historical (retrospective) cohort study with nested case–control analysis. Korea has a universal single-payer health insurance system that covers the entire Korean population (about 50 million people) [14]. The Health Insurance Review and Assessment Service (HIRA) is part of the Korean National Federation of Medical Insurance that reviews medical claims. Upon data requesting and review process, fully anonymized claims data are open to researchers with academic or public policy purposes. The HIRA database contains hospital visits, diagnoses, medical procedures, prescriptions, demographics, and mortality data. Diagnoses in each hospital visit are recorded according to the International Classification of Diseases, 10th revision (ICD-10) codes. Detailed information on the HIRA database is available in previous publications and a large number of epidemiologic studies based on the HIRA database have been published [15, 16]. This study was approved by the Institutional Review Board of Yongin Severance Hospital (9-2020-0116) and the requirement for informed consent was waived owing to the retrospective study design using fully anonymized data.

### Study Participants

From the HIRA database, we established a cohort of adult patients (≥ 20 years) who had received primary diagnosis of RAO between January 2007 and March 2020. Both central (CRAO; ICD-10 code “H34.1”) and branch (BRAO; ICD-10 code of “H34.2”) RAO patients were included [15, 17]. The index date of cohort enrolment was defined as the date of initial diagnosis. To only include patients with newly diagnosed RAO, we set a wash-out period (2007) and excluded those diagnosed with retina vascular occlusion (“H34”) during the washout period. We also excluded patients who had a history of prior cardiovascular disease (ischemic heart diseases: “I20–25”, stroke: “I60–64”, “I69”, carotid artery stent, carotid endarterectomy, coronary stent insertion, coronary artery bypass graft) before the index date. We excluded patients who had primary outcome within a period of less than 7 days of RAO diagnosis. This is because RAO and ischemic stroke often occur simultaneously and asymptomatic ischemic stroke is commonly detected in brain imaging studies following RAO, and it is hard to distinguish whether it occurred after RAO or concurrently. Also, patients with less than 7 days of follow-up (early mortality or loss to follow-up) were excluded, as too short follow-up was insufficient to evaluate the effect of the drug after RAO.

### Study Outcome

The primary outcome was defined as a composite of stroke and myocardial infarction (MI) that occurred after RAO. Development of stroke was determined as admission to hospital with a primary diagnosis of ICD-10 code “I60–63” and brain computed tomography or magnetic resonance imaging performed during the admission [16]. MI was defined as admission with a primary diagnosis of ICD-10 code “I21” [16]. If multiple outcomes occurred in a patient during the follow-up period, the first occurrence was used as the primary outcome. The diagnostic accuracy of stroke (> 80%) and MI (73–93%) based on the health claims database in Korea have been reported to be sufficiently high [18, 19]. Study patients were followed up until the development of primary outcome, loss of participant eligibility, death, or June 30, 2021, whichever occurred the earliest.

### Selection of Cases and Controls

To construct a nested case–control study, we defined cases as patients who had a primary outcome during the follow-up period after RAO. For each case, we sampled two controls with replacement from the cohort, who were event-free and at risk at the time of primary outcome occurrence of their matched case, by incidence density sampling [20]. Controls were required to be of the same sex, age (± 1 year is allowed), and insurance type as their matched case and to be have same comorbidities (hypertension, diabetes mellitus, atrial fibrillation, and renal disease) at the time of case occurrence. In a secondary outcome analysis for individual outcomes, the cases were selected only from those who suffered the outcome of interest first.

### Assessment of Medications

In a nested case–control study, treatments with statin and antiplatelets after RAO were evaluated at the time of primary outcome in cases and at the matched time in controls. Drug administration during the longitudinal follow-up period typically has a time-varying feature. In Korea, statins should be prescribed by a physician. Therefore, medication-related information (drug name, dosage, and duration) is available in the HIRA claims database. In a nested case–control study, treatments with statin (atorvastatin, fluvastatin, lovastatin, pitavastatin, pravastatin, rosuvastatin, and simvastatin) and antiplatelets (aspirin, clopidogrel, ticlopidine, prasugrel, ticagrelor, triflusal, and cilostazol) after RAO were evaluated at the time of primary outcome in cases and at the matched time in controls. Along with statins and antiplatelets treatment after RAO, we evaluated premorbid statin use and premorbid antiplatelet use, which were determined as exposure to the medications within 1 week before the date of RAO diagnosis.

### Other Covariates

We collected information on demographics (age and sex), type of insurance (national health insurance and medical aid from the government), type of RAO (CRAO and BRAO), and comorbidities from the HIRA database. The public healthcare system in Korea is a two-tiered system, national health insurance and medical aid. The Korean medical aid program provides health service at free or reduced-cost to low-income families and individuals. The remaining population are covered by the national health insurance. Identified comorbidities were hypertension, diabetes mellitus, atrial fibrillation, and renal disease. Hypertension or diabetes mellitus was determined if the patients received anti-hypertensive or anti-diabetic medications with the corresponding diagnostic codes (ICD-10 code “I10–I13” and “I15” for hypertension, ICD-10 code “E08–E11” or “E13–E14” for diabetes mellitus). Atrial fibrillation was identified by the presence of specific ICD-10 code “I48.” Renal disease was determined by the presence of related diagnostic codes (ICD-10 codes “N17–N19”, “E08.2”, “E10.2”, “E11.2”, “E13.2”, or “I12–I13”) or claims of hemodialysis or peritoneal dialysis. The presence of comorbidities was determined as whether the diagnosis was present until the time of primary outcome in cases or the matched time point in controls.

### Statistical Analysis

Baseline characteristics are expressed as means ± standard deviations for continuous variables and numbers (%) for categorical variables. We performed conditional logistic regression with the matched case–control groups to estimate the odds ratio (OR) and 95% confidence interval (CI) for primary outcome. Adjustments were performed for RAO type, premorbid statin and premorbid antiplatelet use before RAO, and treatment with statins and antiplatelets after RAO. We also investigated the risk for primary outcome according to the cumulative exposure duration of statin, which is defined as the sum of days covered by statins between RAO diagnosis and the time of primary outcome in case or the matched time in control. The cumulative exposure to statins after RAO was subdivided into four categories: ≤ 90 days, 91–365 days, 1–2 years, and > 2 years. Secondary outcome analyses were performed by constructing individual conditional logistic regression models with cases outcome of ischemic stroke (ICD-10 code “I63”), hemorrhagic stroke (ICD–10 code “I60–I62”), and MI (ICD-10 code “I21”), which constituted the primary outcome. We also performed subpopulation analysis according to RAO type and subgroup analysis according to sex, age, insurance type, hypertension, diabetes mellitus, atrial fibrillation, and renal disease. All statistical analyses were performed by SAS (version 9.4.2; SAS Institute), and R (version 3.5.1; R Foundation for Statistical Computing; http://www.R-project.org/).

## Results

### Characteristics of the RAO Patients

**Fig. 1:**
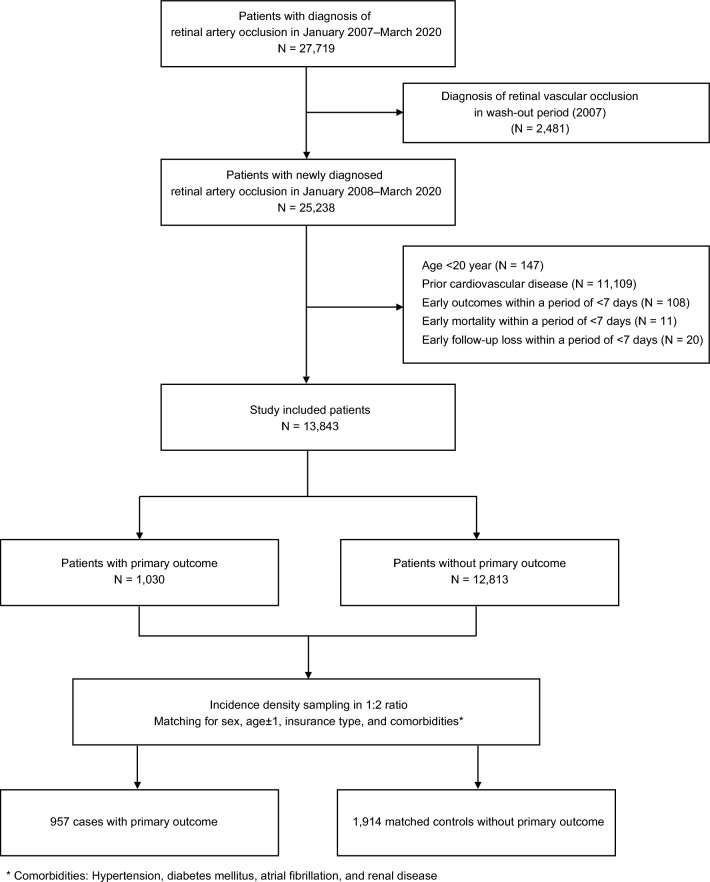
Flowchart of study participants according to inclusion and exclusion criteria. *HIRA* health insurance and review assessment

Between January 2007 and March 2020, 27,719 patients were diagnosed with RAO. After excluding 13,876 patients according to the criteria, we found a total of 13,843 patients newly diagnosed with RAO between January 2008 and March 2020 (Fig. 1). In a cohort with 13,843 RAO patients, the mean age at RAO diagnosis was 60.1 ± 14.2 years, and 57.1% of patients were male (Table 1). Among them, 5,005 (36.2%) and 8,838 (63.8%) were diagnosed with CRAO and BRAO, respectively. Upon evaluating premorbid statin use, there were 14.6% of patients took premorbid statins before RAO. Immediately after RAO diagnosis, the proportion of patients taking statin increased by about 10%, and by the end of follow-up, it further increased to about 40% (Supplementary Fig. S1).

**Table 1:** Baseline characteristics of patients in a nationwide cohort with newly diagnosed retinal artery occlusion

Variables	Study participants			*P*^a^
	Total (n = 13,843)	Type		
		Central retinal artery occlusion (n = 5005)	Branch retinal artery occlusion (n = 8838)	
Sex, male	7902 (57.1)	2968 (59.3)	5061 (57.3)	< 0.001
Age, year^b^	60.1 ± 14.2	61.2 ± 14.7	59.5 ± 13.9	< 0.001
Insurance type				0.009
Health insurance	13,179 (95.2)	4733 (94.6)	8446 (95.6)	
Medical aid	664 (4.8)	272 (5.4)	392 (4.4)	
Comorbidity^b^				
Hypertension	6803 (49.1)	2,456 (49.1)	4347 (49.2)	0.911
Diabetes mellitus	2802 (20.2)	1114 (22.3)	1688 (19.1)	< 0.001
Atrial fibrillation	276 (2.0)	102 (2.0)	174 (2.0)	0.829
Renal disease	1062 (7.7)	398 (8.0)	664 (7.5)	0.369
Premorbid medication^c^				
Statin	2020 (14.6)	681 (13.6)	1339 (15.2)	0.014
Antiplatelet	1813 (13.1)	682 (13.6)	1131 (12.8)	0.173
Year of RAO diagnosis				< 0.001
2008–2011	4825 (34.9)	1890 (37.8)	2935 (33.2)	
2012–2015	4315 (31.2)	1487 (29.7)	2828 (32.0)	
2016–2020	4703 (34.0)	1628 (32.5)	3075 (34.8)	

Data are number (%) or mean ± standard deviation

*RAO* retinal artery occlusion

^a^*P* value is derived from independent t-test or Chi’s square test according to type of retinal artery occlusion

^b^Age and comorbidity at the diagnosis of retinal artery occlusion

^c^Whether the patient had exposure to the medications within 1 week before the date of RAO diagnosis

### Primary Outcome Risk After RAO

**Fig. 2:**
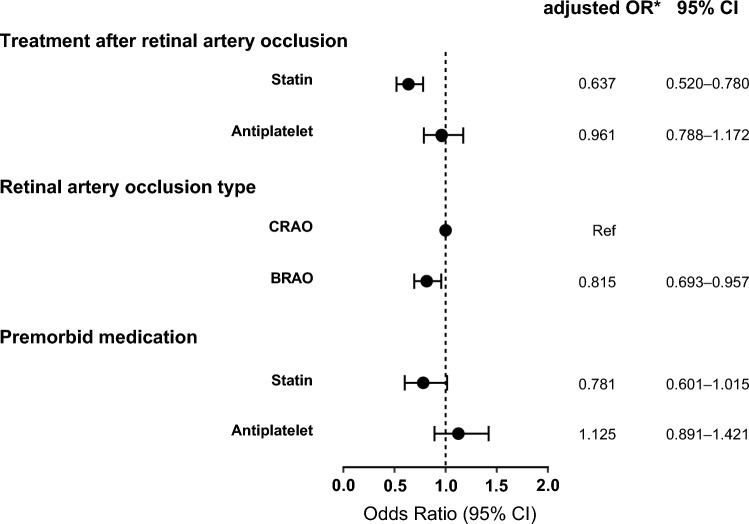
Effects of statins on the risk for primary outcome after retinal artery occlusion. Data are obtained from multivariable conditional logistic regression analysis with the case–control dataset matched for sex, age, insurance type, hypertension, diabetes mellitus, atrial fibrillation, and renal disease. The primary outcome is a composite of stroke and myocardial infarction. *BRAO* branch retinal artery occlusion, *CI* confidence interval, *CRAO* central retinal artery occlusion, *OR* odds ratio

During the mean follow-up period of 6.4 ± 3.7 years after RAO, there were 1,030 patients (7.4%) had a primary outcome in the cohort with 13,843 patients with RAO. With the use of a nested case–control approach, we finally selected 957 cases with primary outcome and 1,914 matched controls without primary outcome by incidence density sampling with the cohort of 13,843 RAO patients (Fig. 1). Table 2 showed the clinical characteristics of the included cases and controls. After the matching process, sex, age, insurance type, presence of hypertension, diabetes mellitus, atrial fibrillation, and renal disease were controlled between case and control groups. The mean time from RAO diagnosis to primary event in cases was 3.6 ± 3.0 years. In the control group, there were more patients treated with statins after RAO than in the case group (37.8% in the control group, 27.6% in the case group, P < 0.001). In the multivariable conditional logistic regression, treatment with statin was associated with lower risk of the primary outcome (adjusted OR, 0.637; 95% CI 0.520–0.780) (Fig. 2). Unlike statin treatment, antiplatelet treatment was not associated with risk for primary outcome (adjusted OR, 0.961; 95% CI 0.788–1.172). When we additionally evaluated the risk according to the cumulative exposure duration of statin treatment, the longer the patient received statins after RAO, the lower the risk of primary outcome. Compared to cumulative statin exposure of ‘ ≤ 90 days’, adjusted OR [95% CI] for’91–365 days’, ‘1–2 years’, ‘ > 2 years’ were 0.713 [0.548–0.929], 0.675 [0.499–0.913], 0.501 [0.391–0.641], respectively.

**Table 2:** Characteristics of case and controls in the nested-case control study

Variable	Study participants		Crude odds ratio (95% CI)	*P*
	Case	Control		
	(n = 957)	(n = 1914)		
Sex, male	648 (67.7)	1296 (67.7)	Matched	–
Age, year^a^	65.1 ± 11.4	65.1 ± 11.4	Matched	–
Time from RAO diagnosis to date of primary outcome in cases or matched date in controls, years	3.6 ± 3.0	3.6 ± 3.0	Matched	–
Insurance type			Matched	
Health insurance	910 (95.1)	1820 (95.1)		
Medical aid	47 (4.9)	94 (4.9)		
Retinal artery occlusion type				
CRAO	400 (41.8)	721 (37.7)	1 (ref)	–
BRAO	557 (58.2)	1193 (62.3)	0.841 (0.718–0.986)	0.032
Comorbidities				
Hypertension	727 (76.0)	1,454 (76.0)	Matched	–
Diabetes mellitus	327 (34.2)	654 (34.2)	Matched	–
Atrial fibrillation	56 (5.9)	112 (5.9)	Matched	–
Renal disease	167 (17.5)	334 (17.5)	Matched	–
Premorbid medication				
Statin	117 (12.2)	329 (17.2)	0.645 (0.509–0.818)	< 0.001
Antiplatelet	173 (18.1)	341 (17.8)	1.020 (0.823–1.264)	0.855
Treatment after retinal artery occlusion				
Statin	264 (27.6)	724 (37.8)	0.600 (0.502–0.716)	< 0.001
Antiplatelet	306 (32.0)	676 (35.3)	0.847 (0.712–1.007)	0.061
Cumulative statin exposure after retinal artery occlusion				
≤ 90 days	623 (65.1)	1,064 (55.6)	1 (ref)	–
91–365 days	106 (11.1)	238 (12.4)	0.713 (0.548–0.929)	0.012
1–2 years	80 (8.4)	189 (9.9)	0.675 (0.499–0.913)	0.011
> 2 years	148 (15.5)	423 (22.1)	0.501 (0.391–0.641)	< 0.001

Data are number (%) or mean ± standard deviation. Odds ratio [95% CI] and *P* value are derived from conditional logistic regression analysis

*BRAO* branch retinal artery occlusion, *CRAO* central retinal artery occlusion, *RAO* retinal artery occlusion

^a^Age at diagnosis of RAO

### Secondary Outcome Analysis

Among 957 cases with the primary outcome, there were patients with stroke in 805 (84.1%; ischemic stroke in 657 cases/hemorrhagic stroke in 148 cases) and those with MI in 152 (15.9%). In the secondary outcome analyses (Supplementary Table S1), statin treatment was mainly associated with reduction of risk for ischemic stroke (adjusted OR, 0.582; 95% CI 0.454–0.747). The risk for hemorrhagic stroke (adjusted OR, 0.725; 95% CI 0.420–1.251), and MI (adjusted OR, 0.938; 95% CI 0.580–1.516) were also reduced with statin treatment, but not statistically significant.

### Sensitivity Analysis

**Fig. 3:**
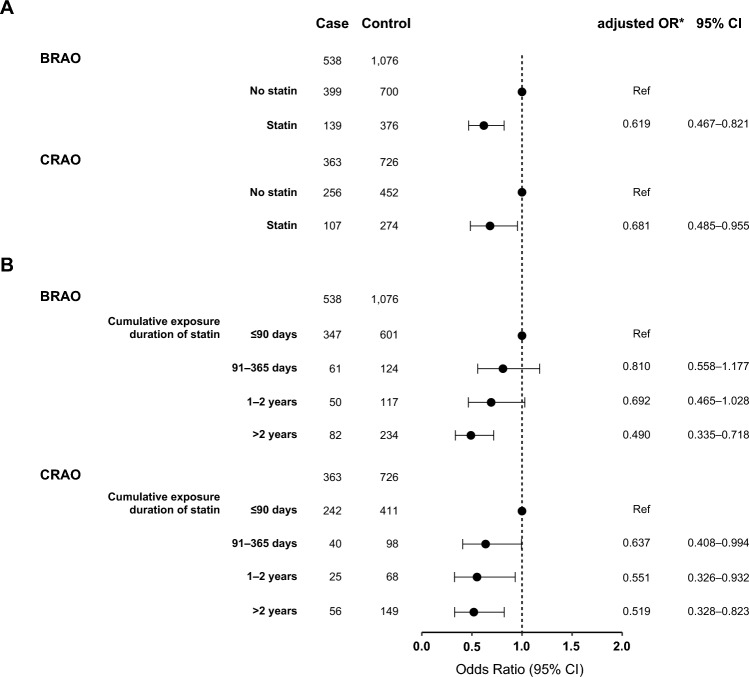
Effects of statins on the risk for primary outcome in subpopulation with branch retinal artery occlusion and central retinal artery occlusion. Data are obtained from multivariable conditional logistic regression analysis with the case–control dataset matched for sex, age, insurance type, hypertension, diabetes mellitus, atrial fibrillation, and renal disease. The primary outcome is a composite of stroke and myocardial infarction. *BRAO* branch retinal artery occlusion, *CI* confidence interval, *CRAO* central retinal artery occlusion, *OR* odds ratio

When we conducted nested case–control analyses with subpopulation with CRAO and BRAO, statin treatment was associated with lower risk for the primary outcome in both CRAO (adjusted OR, 0.681; 95% CI 0.485–0.955) and BRAO (adjusted OR, 0.619; 95% CI 0.467–0.821) (Fig. 3A). The lower risk for primary outcome with longer statin exposure duration was also present in CRAO and BRAO (Fig. 3B). In the subgroup analysis according to sex, age, insurance type, and underlying comorbidities of hypertension, diabetes mellitus, atrial fibrillation, and renal disease, statin treatment was consistently associated with lower risk for primary outcome (Supplementary Fig. S2). When we constructed nested case–control models additionally matched for the year of RAO diagnosis, treatment with oral anticoagulation after RAO, and premorbid statin treatment, the beneficial effect of statin remained significant in each model (Supplementary Fig. S3).

## Discussion

In this nationwide population-based cohort with newly diagnosed RAO patients, about 7.4% experienced future cardiovascular events during the average follow-up period of 6.4 years. Using a nested case–control design, we found that statin treatment after RAO was associated with lower risk for future cardiovascular events. The cardiovascular risk reduction with statin treatment was consistent in both CRAO and BRAO patients. Besides, the longer the duration of statin administration, the lower the cardiovascular risk. About two-thirds of subsequent cardiovascular events after RAO were ischemic stroke, and the preventive effect of statin was mainly attributable to the reduction in ischemic stroke.

The mechanism of RAO, which causes retinal infarction due to RAO, is essentially similar to that of ischemic stroke, which causes brain tissue infarction from cerebral artery occlusion. In the updated definition of stroke published by the American Heart Association/American Stroke Association in 2013, RAO is considered a form of acute ischemic stroke [3]. Just as stroke patients often develop recurrent stroke or MI, RAO patients have a high chance of future cardiovascular events after RAO diagnosis. In a pooled analysis using studies conducted in the USA and Australia, smaller retinal arterioles were associated with an increased risk of cardiovascular mortality [21]. Two nationwide population-based cohort studies to be conducted in Taiwan and Korea have documented a significantly increased stroke risk after RAO. Along with acute stroke, there is evidence that the development of acute coronary events and MI is frequent in RAO patients [9, 10]. Therefore, RAO is considered a forerunner of further cardiovascular and cerebrovascular disease [22].

One reason for the high cardiovascular risk in RAO patients is that they frequently have systemic cardiovascular risk factors and underlying conditions [2]. In this study, about half and a quarter of RAO patients had hypertension and diabetes mellitus, respectively. Specifically, a significant number of RAO patients harbor a severe underlying pathology that requires expedited workup and intensive management [23, 24]. In a study using computed tomography angiography, subclinical coronary artery disease was found in 29% of patients with RAO [25]. In a study investigating cerebrovascular stenosis in RAO patients, 40% of patients had significant atherosclerotic stenosis in the carotid or ophthalmic artery [26]. Retinal microvascular signs have specific associations with large cerebral vessel disease, and large artery atherosclerosis is an independent risk factor for subsequent vascular events [26]. A narrower central retinal arteriole equivalent, focal arteriolar narrowing, and arteriovenous nicking were closely related with further lacunar stroke [27]. A narrower retinal arteriole was also associated with extent and severity of coronary artery disease [28]. Atrial fibrillation, the most common risk factor for cardioembolic stroke, is also a risk factor for RAO, and atrial fibrillation was frequently detected during follow-up after RAO [6]. Additionally, underlying inflammatory conditions and coagulopathy can contribute to the development of both RAO and future cardiovascular events [29].

Cardiovascular disease is a critical illness with a leading cause of mortality worldwide and a major contributor to long-term disability and reduced health-related quality of life. Therefore, prevention of future cardiovascular disease is crucial in RAO management. Current guidelines for prevention of cardiovascular disease strongly recommend statin therapy to patients with atherothrombotic ischemic stroke based on clear evidence that statins reduce the incidence of recurrent stroke, MI, and vascular death [30–34]. Considering the shared risk factors and pathogenesis between ischemic stroke and RAO, and proven prevention role of statins in stroke patients, the prevention effect of statins could be expected in RAO patients as well. However, studies on whether long-term use of statins can reduce cardiovascular events in patients with RAO are lacking.

In this study, we added evidence that statin treatment after RAO was associated with an approximately one-third reduction in cardiovascular risk. Specifically, it was related to reducing ischemic stroke. Statins are widely used drugs for primary and secondary prevention of cardiovascular diseases, such as ischemic stroke and MI [11], as they act as lipid-lowering drugs. However, statins also have multiple pleiotropic (cholesterol-independent) properties that contain anti-inflammatory, anti-thrombotic, blood viscosity-reducing, vasodilating, and endothelium protection effects [35–37]. Statin therapy plays an important role in stabilizing vulnerable plaque and preventing further atherosclerotic plaque progression [38]. The pleiotropic effects of statins might help prevent atrial fibrillation, the most common cause of cardioembolic stroke, and sudden cardiac death [39]. In a recent meta-analysis of atrial fibrillation patients, statin therapy was associated with a 40% reduction in all-cause mortality and a 25% reduction in cardiovascular mortality [40].

Unlike treatment with statins, we did not find an association between treatment with antiplatelets and the risk of further cardiovascular events in RAO patients without previous cardiovascular disease. Indeed, the role of antiplatelets in preventing cardiovascular events in patients with RAO is unclear. In a retrospective cohort study of 9,000 newly diagnosed CRAO patients in Taiwan, the use of aspirin was not associated with a lower risk of ischemic stroke [41]. Real-world data in Denmark showed no protective effect of antithrombotic treatment for future cardiovascular events after RAO [42]. However, we believe that the results of this study don’t mean that the antiplatelets have no role in patients with acute RAO. In our study, several factors may have contributed to the negative finding on antiplatelets. Characteristics of included RAO patients may have influenced the results. We excluded patients with a prior history of cardiovascular disease, considered to be at very high risk. The rate of hypertension is also about half, which is slightly less than that of general stroke patients. The exclusion of patients who had an early event within the first 7 days after RAO, which is the highest risk period of subsequent cardiovascular events, may also have affected the negative result [2, 10]. Recently published scientific statements regard CRAO as a type of ischemic stroke and mention that treatment with antiplatelets is reasonable as pharmacological secondary prevention after CRAO [22]. Further prospective studies on the role of antiplatelets in RAO patients are warranted.

This study has several limitations. This study was a retrospective analysis of the cohort based on the health claims database; therefore, residual confounding by unmeasured factors may exist [43]. We also could not access the underlying causes of RAO, and there might be unmeasured comorbid diseases, such as giant cell arteritis, leading to RAO. Since RAO diagnosis is also based on diagnostic codes in health claims data, there may be a potential problem in misclassification and validation. However, several publications in different countries have identified RAO patients and future cardiovascular events based on a health claims database, suggesting data reliability [15, 41, 44]. We should also acknowledge that there may be differences between the prescription and actual administration of the medications. Since Korea has a relatively ethnically homogeneous population and a public health care system, caution is needed in generalizing the results of this study to other countries. Future studies to identify the effects of statin on RAO patients in other countries will help to generalize the results of this study. Despite these limitations, this nationwide population-based cohort study is meaningful in investigating the role of statins in preventing cardiovascular events, which has not been well-known in RAO patients.

## Conclusions

In this nationwide population-based cohort study of RAO patients, statin treatment was associated with a reduction in long-term cardiovascular outcomes. This effect of statins was observed in both CRAO and BRAO, possibly from the reduction of stroke. The findings of this study need to be reconfirmed through future prospective studies.

### Supplementary Information

Below is the link to the electronic supplementary material.

Supplementary file1 (DOCX 184 KB)

## Data Availability

The data supporting the findings of current study are available from HIRA, but restrictions apply to the availability of the data which were used under license for the current study, and so are not publicly available. To gain access to the data, a completed application form, a research proposal and the applicant’s approval document from the institutional review board should be submitted to and reviewed by the inquiry committee of research support in HIRA (https://opendata.hira.or.kr/or/orb/useGdInfo.do).

## Abbreviations

BRAO: Branch retinal artery occlusion
CI: Confidence interval
CRAO: Central retinal artery occlusion
HIRA: Health Insurance Review and Assessment Service
ICD-10: International Classification of Diseases, 10th revision
MI: Myocardial infarction
OR: Odds ratio
