# Supplementary material for: Statin Treatment on Cardiovascular Risk After Retinal Artery Occlusion: A Historical Cohort Study
Source: J Epidemiol Glob Health. 2023 Aug 12;13(4):685–95. doi: 10.1007/s44197-023-00143-y (PMC10686962; doi:10.1007/s44197-023-00143-y)
Supplement: Supplementary file 1 — Supplementary file1 (DOCX 184 KB) [file 44197_2023_143_MOESM1_ESM.docx]

**SUPPLEMENTAL MATERIALS**

**Supplementary Table S1.** Risk for individual outcome according to treatment after retinal artery occlusion

| Treatment after retinal artery occlusion | All stroke (n=805) | | Ischemic stroke (n=657) | | Hemorrhagic stroke (n=148) | | Myocardial infarction (n=152) | |
| --- | --- | --- | --- | --- | --- | --- | --- | --- |
|  | Adjusted OR (95% CI) | P | Adjusted OR (95% CI) | P | Adjusted OR (95% CI) | P | Adjusted OR (95% CI) | P |
| Statin | 0.600 (0.479–0.752) | <0.001 | 0.582 (0.454–0.747) | <0.001 | 0.725 (0.420–1.251) | 0.248 | 0.938 (0.580–1.516) | 0.793 |
| Antiplatelet | 0.878 (0.704–1.096) | 0.251 | 0.891 (0.698–1.137) | 0.353 | 0.877 (0.509–1.510) | 0.635 | 1.520 (0.952–2.428) | 0.080 |

CI, confidence interval; and OR, odds ratio.

Data are obtained from multivariable conditional logistic regression analyses with the case-control dataset matched for sex, age, hypertension, insurance type, diabetes mellitus, atrial fibrillation, and renal disease.

Adjustments are made to retinal artery occlusion type, premorbid use of statin, and premorbid use of antiplatelet.

**Supplementary Fig S1.** Proportion of patients who received statins before and after being diagnosed with retinal artery occlusion


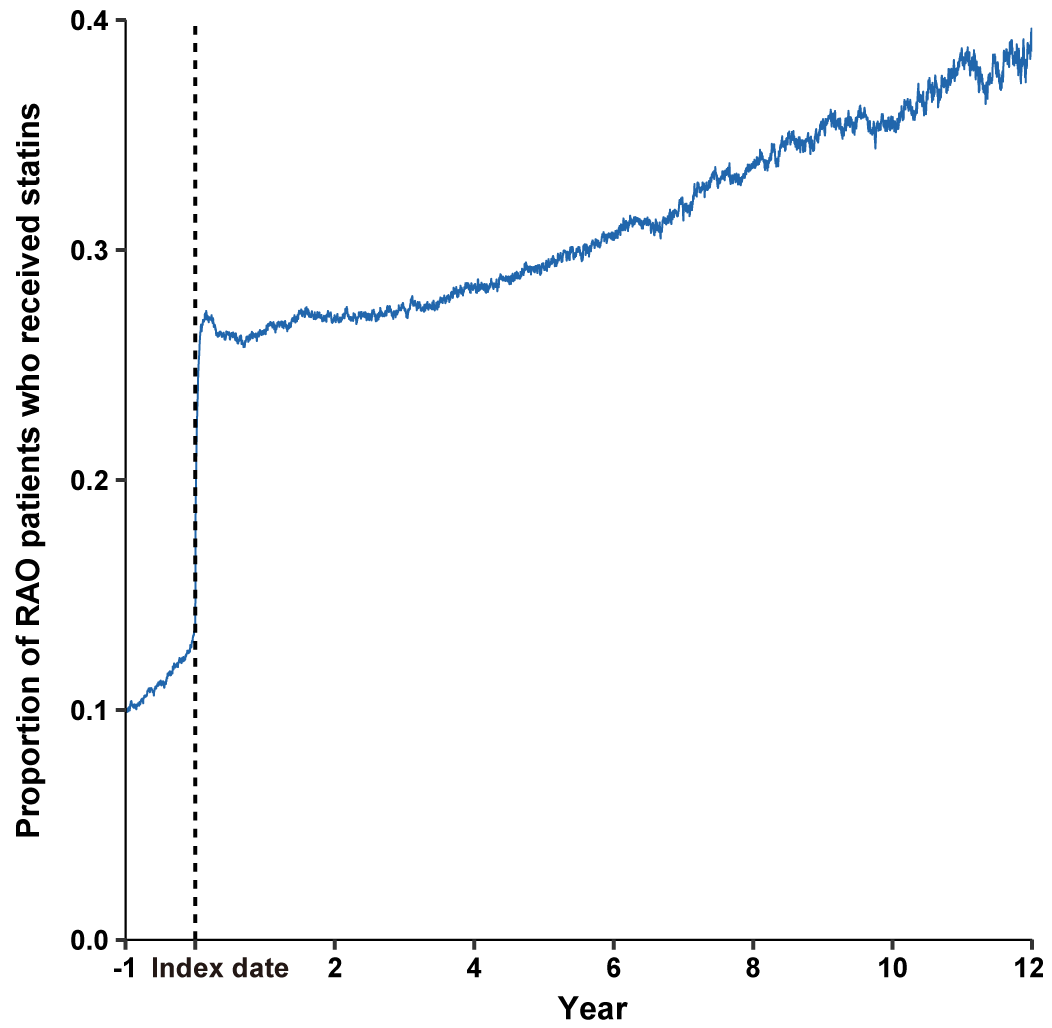


The X-axis indicates the time (year) from the onset of RAO (index date), ranging from 1 year before diagnosis of RAO to the study end date (the development of cardiovascular event, loss of participant eligibility, death, or June 30, 2021, whichever occurred the earliest). The Y-axis indicates the proportion of patients who received medication among those at risk (who were still followed up, excluding those who died, who were included in the outcome, and who were censored) at the time point. Data is derived from the cohort with 13,843 patients newly diagnosed with RAO.

RAO, retinal artery occlusion.

**Supplementary Fig S2.** Results of subgroup analyses for primary outcome according to statin treatment after retinal artery occlusion


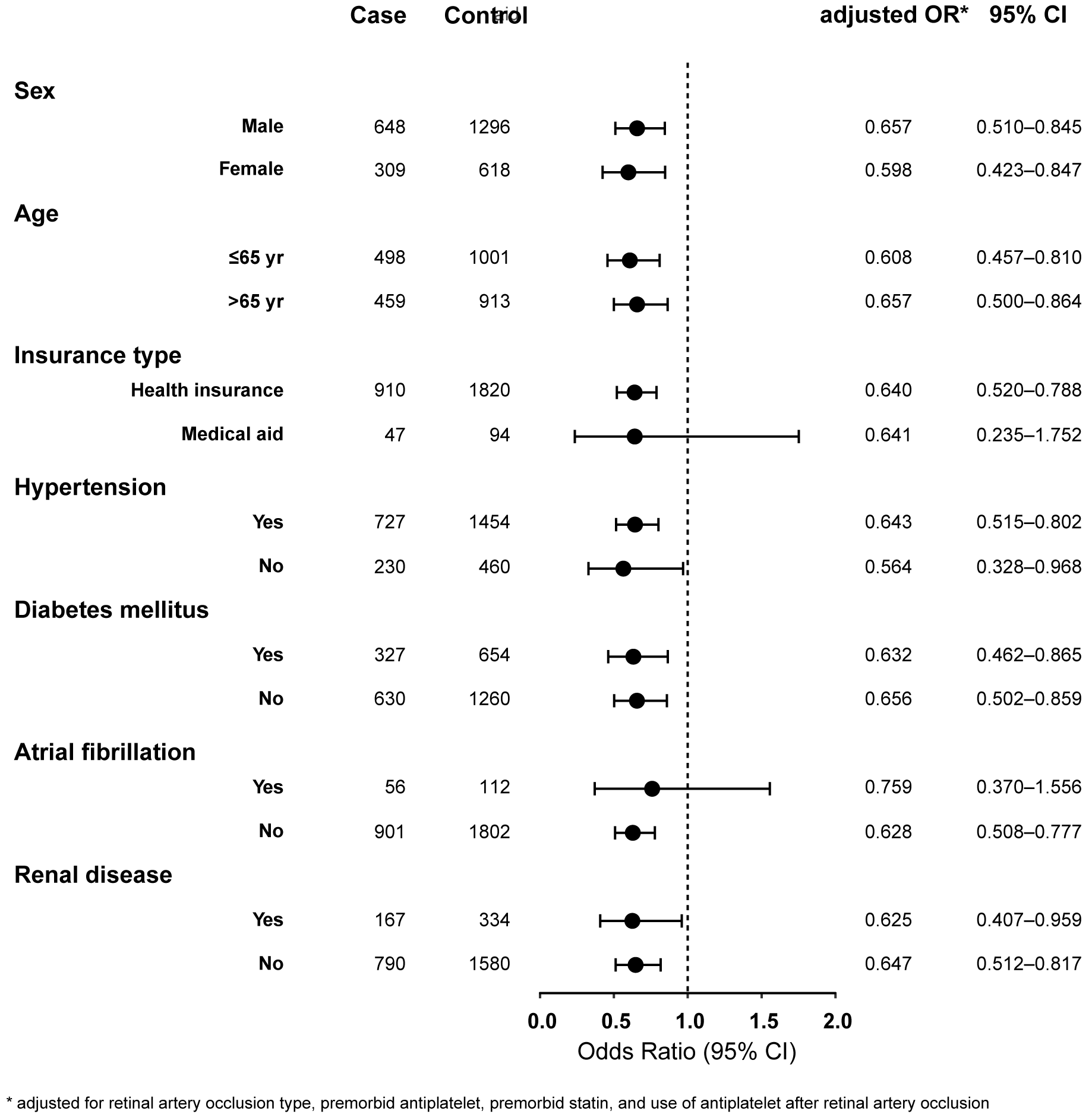
Data includes adjusted odds ratio and 95% confidence interval for statin treatment after retinal artery occlusion, derived from multivariable conditional logistic regression with the matched case-control groups for primary outcome.

CI, confidence interval; and OR, odds ratio.

**Supplementary Fig S3**. Result of the nested case-control studies with dataset additionally matched for the year of retinal artery occlusion diagnosis, oral anticoagulation, and premorbid statin use


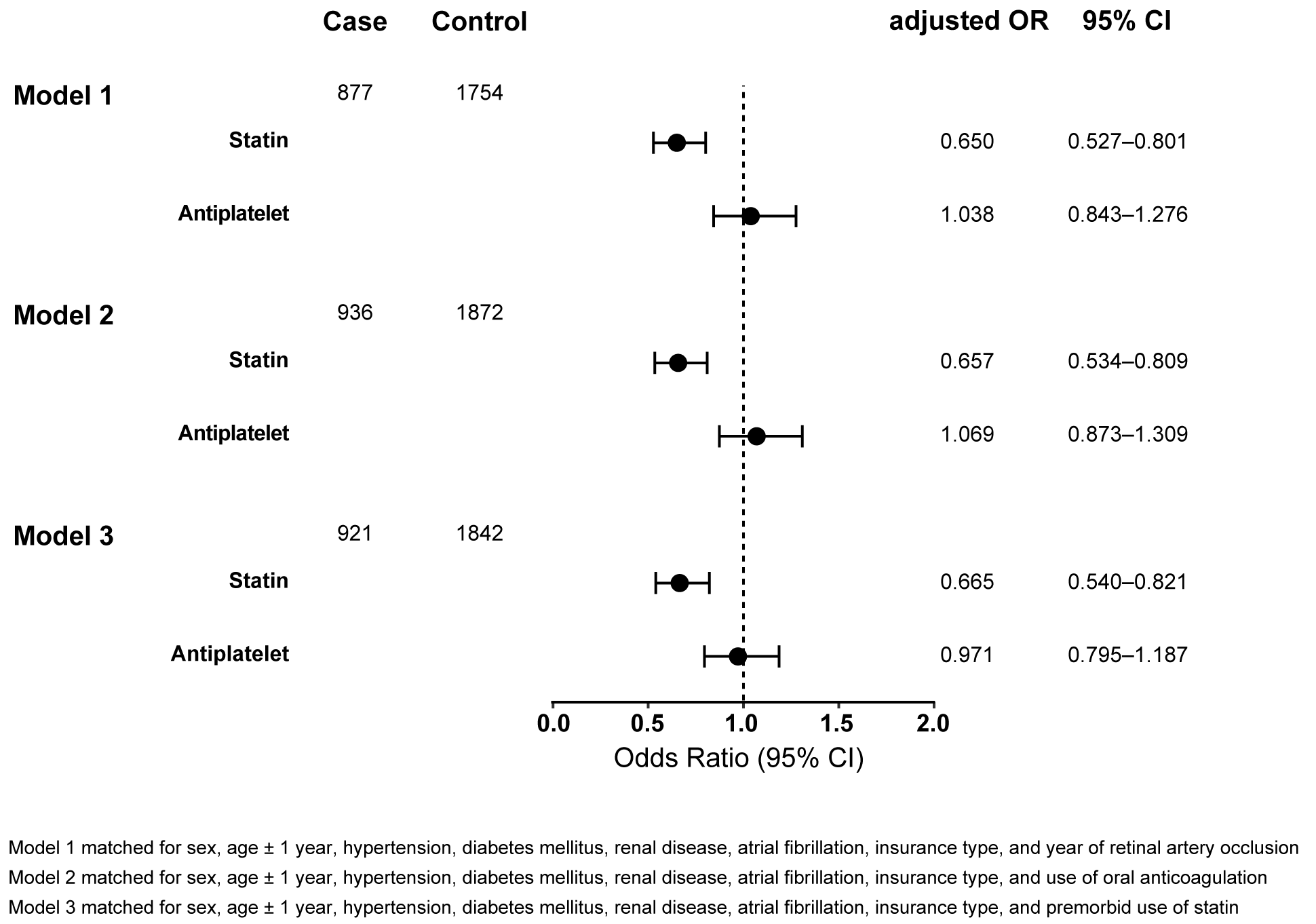


Data shows adjusted odds ratio and 95% confidence interval for statin treatment after retinal artery occlusion, derived from multivariable conditional logistic regression with the matched case-control groups for primary outcome.

Model 1 and 2: adjusted for type of retinal artery occlusion, premorbid statin, and premorbid antiplatelet.

Model 3: adjusted for type of retinal artery occlusion, and premorbid antiplatelet.

CI, confidence interval; and OR, odds ratio.
